# Establishing a National Maternal Morbidity Outcome Indicator in England: A Population-Based Study Using Routine Hospital Data

**DOI:** 10.1371/journal.pone.0153370

**Published:** 2016-04-07

**Authors:** Manisha Nair, Jennnifer J. Kurinczuk, Marian Knight

**Affiliations:** National Perinatal Epidemiology Unit, Nuffield Department of Population Health, University of Oxford, Oxford, United Kingdom; National Institute of Health, ITALY

## Abstract

**Introduction:**

As maternal deaths become rarer, monitoring near-miss or severe maternal morbidity becomes important as a tool to measure changes in care quality. Many calls have been made to use routinely available hospital administration data to monitor the quality of maternity care. We investigated 1) the feasibility of developing an English Maternal Morbidity Outcome Indicator (EMMOI) by reproducing an Australian indicator using routinely available hospital data, 2) the impact of modifications to the indicator to address potential data quality issues, 3) the reliability of the indicator.

**Methods:**

We used data from 6,389,066 women giving birth in England from April 2003 to March 2013 available in the Hospital Episode Statistics (HES) database of the Health and Social care Information centre (HSCIC). A composite indicator, EMMOI, was generated from the diagnoses and procedure codes. Rates of individual morbid events included in the EMMOI were compared with the rates in the UK reported by population-based studies.

**Results:**

EMMOI included 26 morbid events (17 diagnosis and 9 procedures). Selection of the individual morbid events was guided by the Australian indicator and published literature for conditions associated with maternal morbidity and mortality in the UK, but was mainly driven by the quality of the routine hospital data. Comparing the rates of individual morbid events of the indicator with figures from population-based studies showed that the possibility of false positive and false negative cases cannot be ruled out.

**Conclusion:**

While routine English hospital data can be used to generate a composite indicator to monitor trends in maternal morbidity during childbirth, the quality and reliability of this monitoring indicator depends on the quality of the hospital data, which is currently inadequate.

## Introduction

Most women in high resource settings give birth in hospitals or birth centres [1]. In both middle and low resource settings there is a focus on increasing the proportion of facility based deliveries [2–4], although there are concerns over the quality of care received in facilities as the number of women delivering in these settings increases [5,6]. It is thus increasingly important that we identify a meaningful and straightforward means of monitoring the quality of maternity care. Much of the focus has been on process measures such as caesarean section rates, induction of labour rates, neonatal unit admission rates and readmission rates. Monitoring any or all of these measures can be problematic, for example, there is no agreement on what the optimal caesarean delivery rate should be. While it is clear that caesarean delivery can be an essential obstetric intervention to save the life of both mother and baby, it is equally clear that high caesarean delivery rates may represent over-medicalisation and that unnecessary intervention can lead to long-term complications for both mother and baby [7–10]. Similarly monitoring readmission rates may lead to paradoxical responses; for example, readmission rates will decrease if women remain in hospital for longer after giving birth and thus longer stays may result, even though this may not be clinically necessary for, or desired by, most women. Similarly, readmission rates may increase if women are discharged home earlier, even when early discharge is being offered in response to women’s wishes to improve their experience of care.

Outcome measures such as maternal and perinatal mortality are frequently monitored at both health centre and population levels. However as, in particular, maternal death becomes rarer, this becomes less meaningful as it is not very responsive to changes in care quality. The WHO introduced a complex maternal near miss/ morbidity indicator, which requires collection of detailed data including laboratory based parameters indicating organ system dysfunction [11]. However it has been found difficult to implement in some settings [12,13] and particularly, since it requires additional data collection, monitoring represents a burden that many healthcare environments cannot sustain. In response to concerns about data collection burden, many calls have been made to use routinely available hospital administration data in order to monitor outcomes [14–16].

In the UK the Royal College of Obstetrics and Gynaecology have developed a series of “maternity indicators” which include elements such as caesarean section rate [17]. However very few of the indicators are outcomes and the majority are process measures [18]. Importantly, the work identified major concerns over data quality. An approach undertaken by researchers in New South Wales (Australia) was to develop a maternal morbidity outcome indicator, which used a range of outcomes identified in routinely available maternity hospital discharge data to develop a single measure [19]. By including a number of outcomes to develop the single measure, concerns over false negative cases are mitigated, since most women with severe morbidity will have more than one eligible procedure or condition, and thus multiple appropriate codes in the data.

However, it is not clear whether data quality is sufficiently high in England to adopt the Australian approach. The aim of this study was to investigate the feasibility of reproducing the Australian Maternal Morbidity Outcome Indicator using routinely available English hospital maternity data; the impact of modifications to the Indicator to address potential data quality issues as well as known maternal health concerns in the UK; and the reliability of the indicator.

## Materials and Methods

We used routine hospital data from the Hospital Episode Statistics (HES) database of the Health and Social care Information Centre (HSCIC) [20] to develop a single measure of maternal morbidity during childbirth. The HES is a dataset of inpatient, outpatient, and accident and emergency records from all National Health Services (NHS) hospitals in England [20]. In this study, we used a bespoke extract of anonymous inpatient data from childbirth episodes of all 6,389,066 women who gave birth in England from 1 April 2003 to 31 March 2013. Information was available on diagnoses and procedures at the time of childbirth and socio-demographic and pregnancy related characteristics of the women from the recorded hospital data. We constructed an English Maternal Morbidity Outcome Indicator (EMMOI) to measure maternal morbidity outcomes during childbirth using the method employed by Roberts et al to construct the Australian indicator [19] and examined the reliability of the EMMOI as a measure of morbid maternal events during childbirth in England by comparing with evidence from published population-based epidemiological studies.

### Constructing the English Maternal Morbidity Outcome Indicator (EMMOI)

We generated a list of diagnoses and procedures to be included in the composite indicator for England (EMMOI) by reviewing both the initial and final lists of the components of the Australian Maternal Morbidity Outcome Indicator developed by Roberts et al [19] as well as published literature on conditions associated with maternal morbidity and mortality in the UK [21–23]. In the HES data, patient diagnoses are coded using the 10^th^ revision of the International Statistical Classification of Diseases and Related Health Problems (ICD-10) and procedures are coded using the OPCS Classification of Interventions and Procedures used by the NHS hospitals in the UK [24].

### Statistical analysis

We calculated the frequency and rate of the individual morbid events (diagnoses and procedures) in the study population and their annual rates over the period of 10 years in order to examine potential variation in coding practice or data quality. We calculated the incidence rate and 95% confidence intervals (CI) of maternal morbidity outcomes per 1000 women giving birth in England using the EMMOI for each year from April 2003 to March 2013 and conducted tests for linearity to examine their trend over time. We also calculated the percentage change in incidence of maternal morbidity during childbirth in England in 2012–13 compared to 2003–04 and 95% CI.

We examined changes in the maternal sociodemographic and pregnancy characteristics by conducting χ2 tests for differences in proportions and χ^2^ test for trend. This was followed by univariable and multivariable logistic regression analyses to examine whether the change in the odds of maternal morbidity outcomes over the period of 10 years were attributable to the changes in maternal and pregnancy characteristics in England. A core logistic regression model was built including the nine maternal and pregnancy characteristics shown in Table 1. Tests for correlation did not show any significant moderate to strong correlation between the variables. Maternal age was included as a continuous variable. We tested for plausible interactions by fitting interaction terms into the multivariable model followed by likelihood ratio testing (LR-test). No significant interactions were identified. The proportion of missing data was high for all the nine variables included in the model. We did not consider the data to be missing at random and a proxy variable was generated by categorising the missing data as a separate group for each variable. Sensitivity analysis was conducted by redistributing the missing observations into the different categories of the variables. We also performed a complete case analysis. The results of the sensitivity analysis and complete case analysis were not different from that of the main model. All analyses were performed using Stata version 13.1, SE (StataCorp, College Station, TX).

**Table 1:** Change in maternal and pregnancy characteristics in England in 2012–13 compared to 2003–04; Hospital Episode Statistics data England.

Characteristics	2003–04 N = 581,839 Number (%)	2012-13N = 667,729 Number (%)	P-value for the χ^2^ tests for differences in proportions
**Woman’s age (in Years)**			
<20	29 530 (5.1)	26 765 (4.0)	<0.001
20–24	79,333 (13.6)	104,223 (15.6)	
25–29	105,852 (18.2)	160,292 (24.0)	
30–34	126,003 (21.6)	168,864 (25.3)	
35–39	65,701 (11.3)	88,212 (13.2)	
40–44	12,127 (2.1)	20,907 (3.1)	
45–49	495 (0.1)	1,130 (0.2)	
≥50	28 (0.01)	79 (0.01)	
Missing	162,770 (28.0)	97,257 (14.6)	
**Parity**			
Nulliparous	147,326 (25.3)	192,576 (28.8)	<0.001
Multiparous	236,006 (40.6)	287,552 (43.1)	
Missing	198,507 (34.1)	187,601 (28.1)	
**Place of delivery**			
Hospital	346,717 (59.6)	578,829 (86.7)	<0.001
Home	1641 (0.3)	5636 (0.8)	
Others	1256 (0.2)	4784 (0.7)	
Missing	232,225 (39.9)	78,480 (11.8)	
**Change in planned place of delivery**			
Yes	27,473 (4.7)	51,914 (7.8)	<0.001
No	340,903 (58.6)	461,732 (69.1)	
Missing	213,463 (36.7)	154,083 (23.1)	
**Mode of delivery**			
Spontaneous vaginal	283,911 (48.8)	368,464 (55.2)	<0.001
Instrumental vaginal	44,383 (7.6)	71,338 (10.7)	
Elective caesarean	38,555 (6.6)	62,537 (9.4)	
Emergency caesarean	53,289 (9.2)	78,930 (11.8)	
Other procedures	4485 (0.8)	6788 (1.0)	
Missing	157,216 (27.0)	79,672 (11.9)	
**Multiple pregnancy**			
Yes	8459 (1.5)	8586 (1.3)	<0.001
No	478,802 (82.3)	592,729 (88.8)	
Missing	94,578 (16.3)	66,414 (9.9)	
**Ethnicity**			
White	342,209 (58.8)	480,263 (71.9)	<0.001
Mixed	5137 (0.9)	10,017 (1.5)	
Indian	13,117 (2.3)	20,556 (3.1)	
Pakistani	18,911 (3.2)	26,999 (4.0)	
Bangladeshi	8027 (1.4)	9037 (1.4)	
Chinese	1879 (0.3)	4937 (0.7)	
Other Asian	5872 (1.0)	13,927 (2.1)	
Black Caribbean	7190 (1.2)	6444 (1.0)	
Black African	13,202 (2.3)	20,309 (3.0)	
Other black	5034 (0.9)	5751 (0.9)	
Others	11,781 (2.0)	19,140 (2.9)	
Missing	149,480 (25.7)	50,349 (7.5)	
**IMD quintiles**			
I (Least deprived 20%)	98,120 (16.9)	99,116 (14.8)	<0.001
II	97,385 (16.7)	106,052 (15.9)	
III	104,982 (18.0)	123,007 (18.4)	
IV	120,915 (20.8)	151,019 (22.6)	
V (Most deprived 20%)	157,134 (27.0)	185,136 (27.7)	
Missing	3303 (0.6)	3399 (0.5)	
**Place of residence**			
Urban	491,743 (84.5)	573,793 (85.9)	<0.001
Town/ Fringe	43,749 (7.5)	46,824 (7.0)	
Rural	44,092 (7.6)	43,713 (6.6)	
Not in England	67 (0.01)	1372 (0.2)	
Missing	2188 (0.4)	2027 (0.3)	

IMD—Index of multiple deprivation

## Results

**Fig 1:**
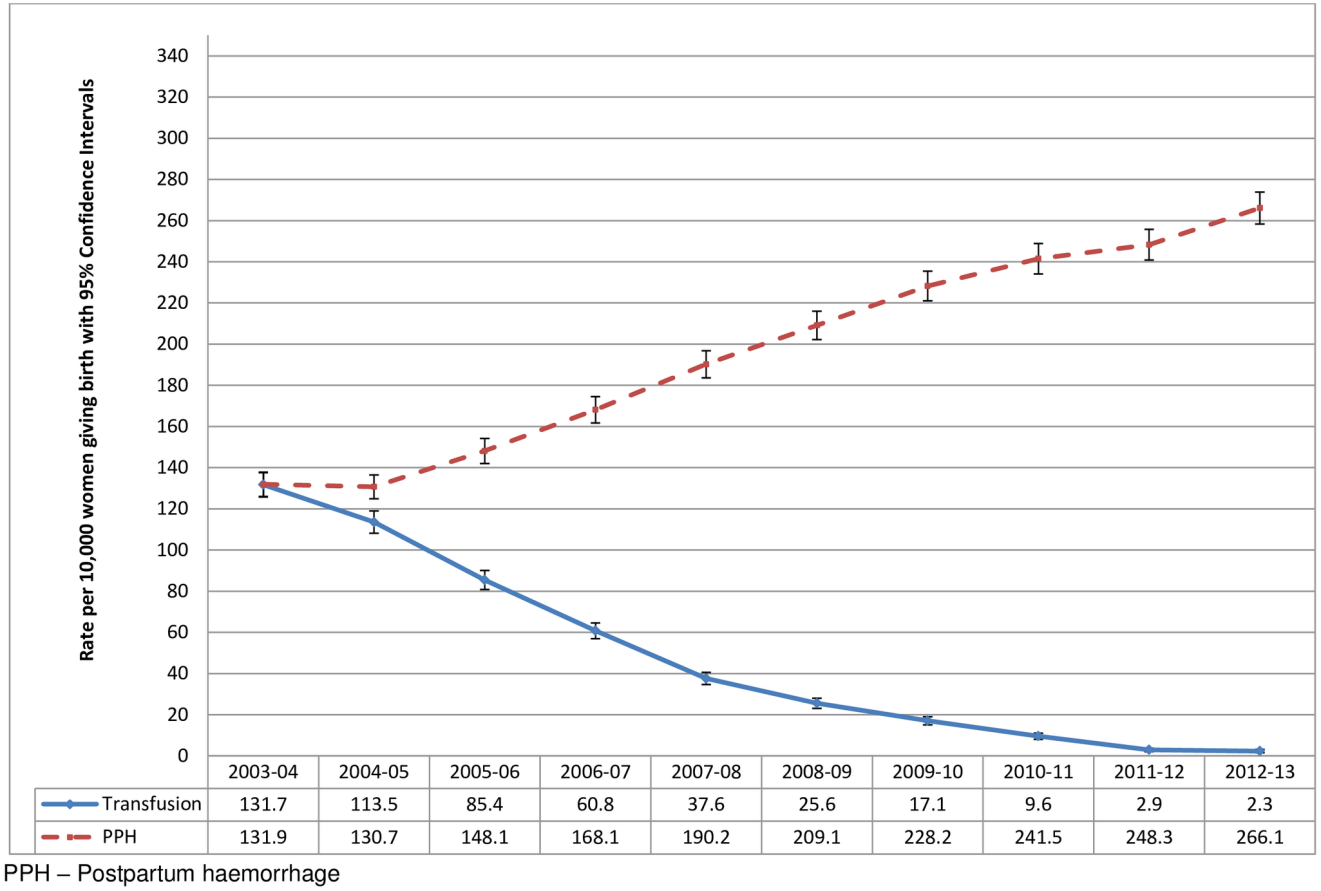
Comparison of the estimated rates of transfusion of blood and blood products and postpartum haemorrhage among women giving birth in England from 2003 to 2013; Hospital Episode Statistics data England.

**Fig 2:**
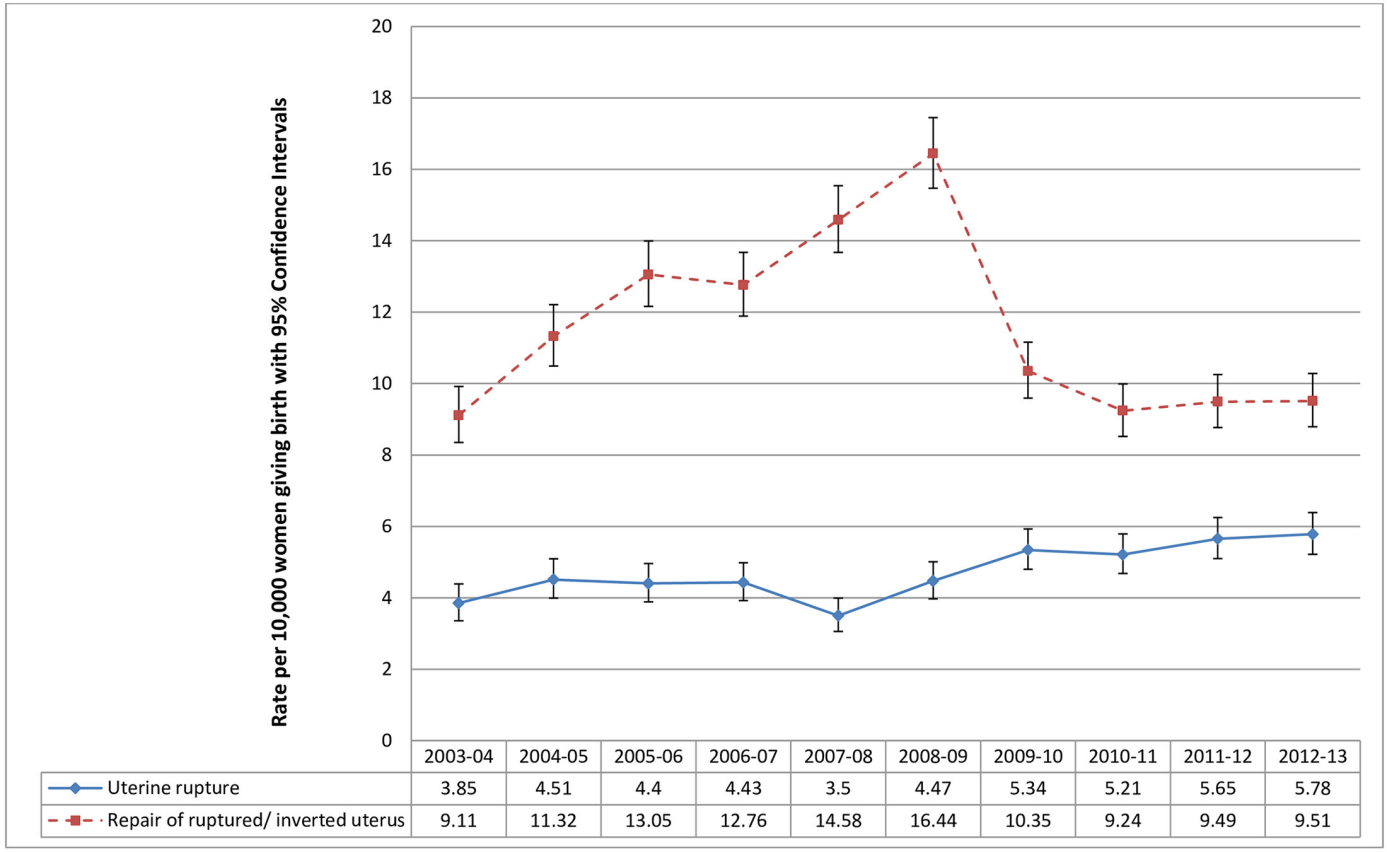
Comparison of the estimated rates of uterine rupture and repair of ruptured or inverted uterus among women giving birth in England from 2003 to 2013; Hospital Episode Statistics data England.

The composite indicator, EMMOI, included all 14 diagnoses and nine out of 10 procedures from the final list of components of the Australian indicator as well as sepsis, eclampsia and cerebral venous thrombosis, which are important causes of maternal morbidity and mortality in the UK [21–23]. Diagnostic codes for sepsis, eclampsia and thrombosis were initially included in the Australian indicator, but were later removed at the stage of refining as these were poorly defined in the Australian population health datasets used to develop the indicator. Each diagnosis and procedure was examined before inclusion in the final EMMOI by calculating the incidence rate and examining the trend over time from 2003–13. We observed a 60-fold decrease in the estimated rate of transfusion of blood and blood products from 132 per 10,000 childbirth episodes in 2003–04 to 2 per 10,000 childbirth episodes in 2012–13, in contrast to a more than doubling of the estimated rate of postpartum haemorrhage (PPH) over the same period of time (Fig 1). Based on this observation we did not consider the transfusion or PPH data to be reliable and thus these codes were not included in the final EMMOI. We also observed a mismatch between diagnosis of ‘uterine rupture’ and the procedure ‘repair of ruptured or inverted uterus’ with substantial fluctuation in the rates across the years (Fig 2). The overall incidence of uterine rupture estimated using the diagnosis code was closer to the rate estimated by a population-based study in the UK (1.9 per 10,000 maternities, 95% CI = 1.6 to 2.2) [25], thus we included the diagnosis code and excluded the procedure code from the final indicator. The final list of diagnoses included in the EMMOI were acute abdomen, acute renal failure, acute psychosis, cardiac arrest/ failure or infarction, cerebral oedema or coma, disseminated intravascular coagulopathy, cerebrovascular accident, major complications of anaesthesia, obstetric embolism including amniotic fluid embolism, shock, sickle cell anaemia with crisis, status asthmaticus, status epilepticus, uterine rupture, eclampsia, sepsis, and cerebral venous thrombosis. The procedures included in the EMMOI were assisted ventilation including tracheostomy, curettage in combination with a general anaesthetic, dialysis, evacuation of haematoma, hysterectomy, procedures to reduce blood flow to uterus, re-closure of disrupted caesarean section wound, repair of bladder or cystostomy, and repair of intestine.

**Fig 3:**
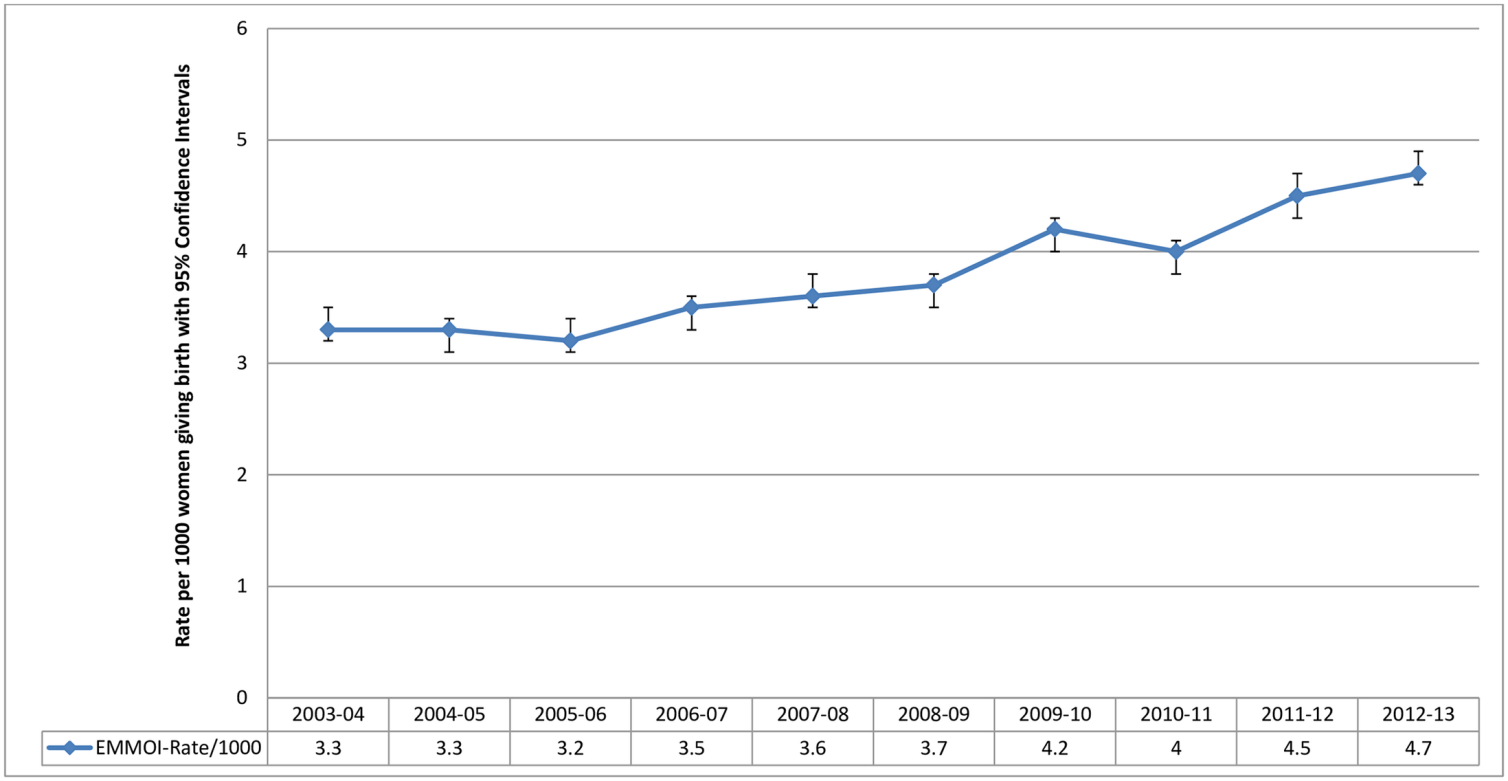
Trends in maternal morbidity outcomes during childbirth in England from 2003–04 to 2012–13 using the EMMOI; Hospital Episode Statistics data England.

Of the 6,389,066 women giving birth in England from 2003 to 2013, the EMMOI classified 24,427 women as having experienced one or more maternal morbidity during childbirth. There was an increase in the annual rate of maternal morbidity outcomes from 2003 to 2013 (Fig 3) with a linear trend (p<0.001), even after accounting for the changes in maternal and pregnancy characteristics overtime (shown in Table 1). The crude and adjusted odds ratios for each year compared to 2003–04 are shown in Table 2. The estimated rates using the EMMOI show an overall 29% (95% CI 25% to 33%) absolute increase in the incidence of maternal morbidity during childbirth in England in 2012–13 compared to 2003–04.

**Table 2:** Maternal morbidity outcomes during childbirth in England from 2003 to 2013; Hospital Episode Statistics data England.

Year	Cases (%) N = 24,427	Controls (%) N = 6,364,639	Unadjusted OR (95% CI)	Adjusted OR**(95% CI)	% change in incidence rate 2012–13 compared to 2003–04 (95% CI)*
2003–04	1944 (0.33)	579,895 (99.67)	1 (ref)	1 (ref)	+29.2 (+25.1 to +33.0)
2004–05	1960 (0.33)	594,144 (99.67)	0.98 (0.92 to 1.05)	0.97 (0.91 to 1.04)	
2005–06	1974 (0.32)	608,804 (99.68)	0.97 (0.91 to 1.03)	0.93 (0.88 to 0.99)	
2006–07	2183 (0.35)	628,135 (99.65)	1.04 (0.98 to 1.10)	0.99 (0.93 to 1.05)	
2007–08	2351 (0.36)	645,798 (99.64)	1.09 (1.02 to 1.15)	1.01 (0.95 to 1.07)	
2008–09	2407 (0.37)	651,071 (99.63)	1.10 (1.04 to 1.17)	1.05 (0.99 to 1.12)	
2009–10	2748 (0.42)	654,195 (99.58)	1.25 (1.18 to 1.33)	1.20 (1.13 to 1.28)	
2010–11	2677 (0.40)	668,565 (99.60)	1.19 (1.13 to 1.27)	1.15 (1.08 to 1.22)	
2011–12	3032 (0.45)	669,454 (99.55)	1.35 (1.28 to 1.43)	1.32 (1.24 to 1.40)	
2012–13	3151 (0.47)	664,578 (99.53)	1.41 (1.34 to 1.50)	1.37 (1.29 to 1.45)	

**Adjusted for the nine maternal and pregnancy characteristics shown in Table 1;

*p-value for χ2 test for trend across the years was <0.001

**Fig 4:**
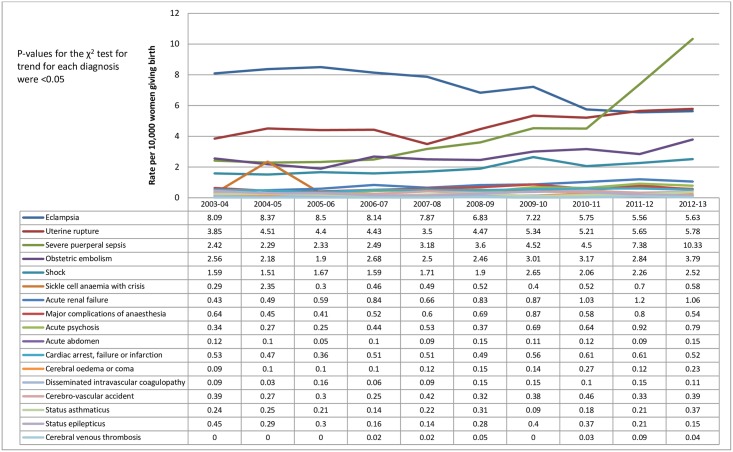
Annual rate per 10,000 women of the individual morbid events/ diagnoses included in the EMMOI (2003 to 2013); Hospital Episode Statistics data England.

**Fig 5:**
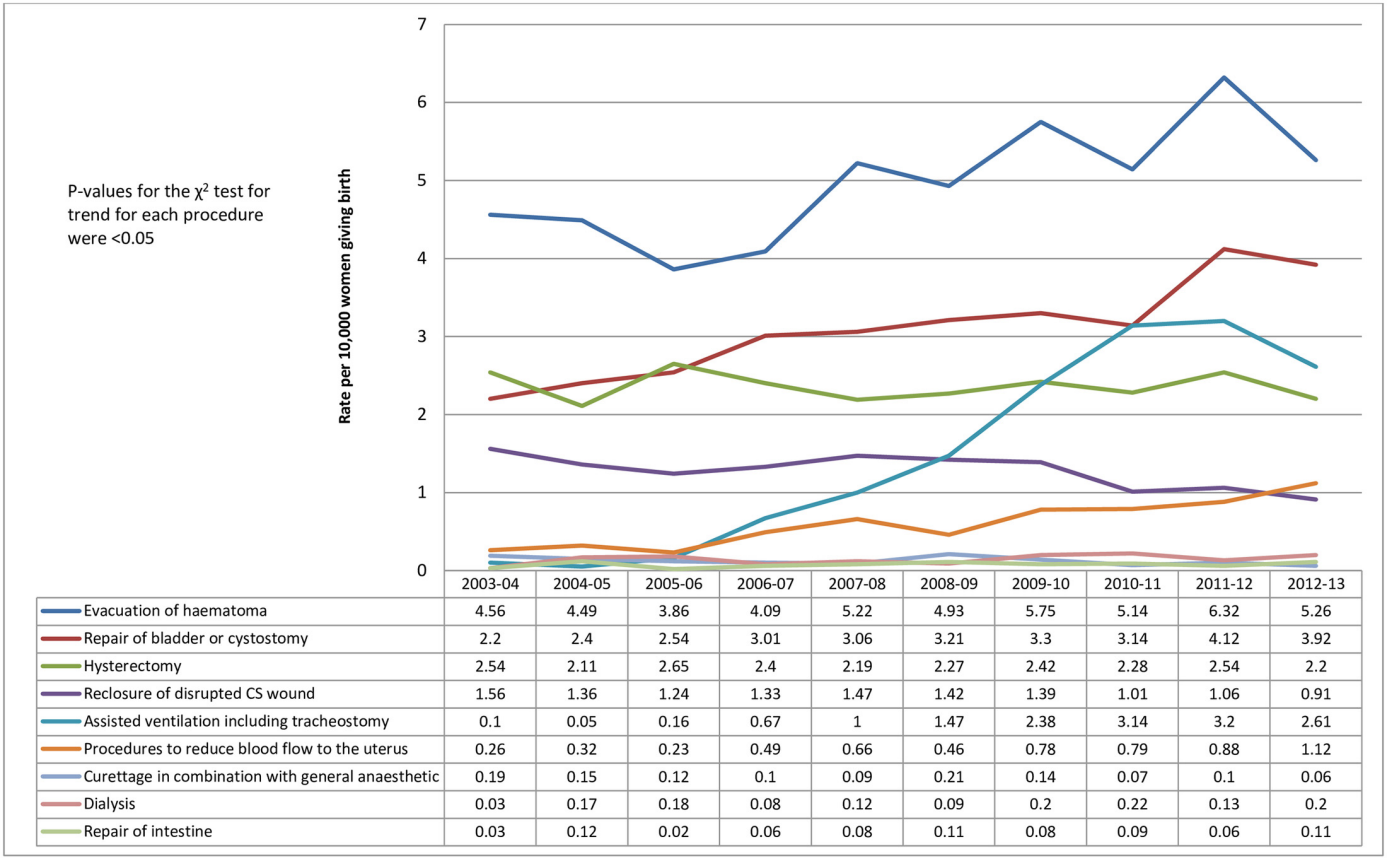
Annual rate per 10,000 women of the individual procedures included in the EMMOI (2003 to 2013); Hospital Episode Statistics data England.

The overall frequency and rate of the individual component diagnoses and procedures (along with their codes) included in the EMMOI are shown in Table 3 and their trends across 10 years are shown in Figs 4 and 5. The most common diagnosis coded was eclampsia (7.2 per 10,000 deliveries; 95% CI 6.9 to 7.4) followed by evacuation of haematoma (4.9 per 10,000 deliveries; 95% CI 4.8 to 5.2), uterine rupture (4.8 per 10,000 deliveries; 95% CI 4.6 to 5.0) and severe puerperal sepsis (4.4 per 10,000 deliveries; 95% CI 4.2 to 4.6). While the rate of eclampsia decreased over the 10 years, the rates of other morbid events either increased or remained unchanged, with notable increases being for sepsis and uterine rupture (Fig 4), assisted ventilation including tracheostomy and repair of bladder or cystostomy (Fig 5).

**Table 3:** Comparing the rate of individual morbid events constituting the EMMOI with UK rates from published studies and rates of morbid events constitution the Australian indicator.

Indicators included in the English MMOI	Codes	Number of women (Hospital Episode Statistics data)	Rate per 10,000 women giving birth (95% Cl), Hospital Episode Statistics data	Available incidence rates per 10,000 women giving birth (95% Cl)* in the UK from published studies and reports	Incidence of morbid events constituting the Australian indicator (Roberts et al, 2009)Rate/ 10,000	Comparable with—the UK or Australian indicator or both/ Not comparable
**Morbid events/ diagnosis (ICD-10 codes)**						
Acute abdomen	K35, K37, K65.0, K65.9, N73.3, N73.5	69	0.11 (0.01 to 0.14)	Not available	0.36	Comparable with the Australian indicator
Acute renal failure	O90.4, N17, N19, N99.0	518	0.81 (0.74 to 0.88)	Not available	2.10	Not comparable
Acute psychosis	F23, F53.1	340	0.53 (0.48 to 0.59)	10 per 10,000 pregnant women in the UK [26]	0.58	Comparable with the Australian indicator
Cardiac arrest/ failure or infarction	O89.1, O74.2, O90.3, I21, I42, I43, I46, I50, J81	332	0.52 (0.47 to 0.58)	0.33 per 10,000 pregnant women in the UK [27]. Myocardial infarction alone 0.07 per 10,000 maternities in the UK (95% CI 0.05 to 0.11) [28]	4.49	Comparable with the UK
Cerebral oedema or coma	G93.6, R40.2	91	0.14 (0.12 to 0.18)	Not available	Not reported	Not known
Disseminated Intravascular Coagulopathy	D65	70	0.11 (0.01 to 0.14)	Not available	Not reported	Not known
Cerebrovascular accident	I60, I61, I62, I63, I64	225	0.35 (0.31 to 0.40)	Antenatal stroke 0.15 per 10,000 maternities in the UK (95% CI 1.0 to 2.1) [29]. Postpartum stroke 0.16 per 10,000 maternities in the UK (95% CI 0.11 to 0.23) [29]. Overall 0.31 per 10,000 maternities in the UK (95% CI 0.24 to 0.40) [29]	0.32	Comparable with the UK and Australian indicator
Major complications of anaesthesia	O74.0, O74.1, O74.2, O74.3, O74.9, O89.0, O89.1, O89.2, O29.0, O29.1, O29.2	392	0.61 (0.55 to 0.68)	Serious non-fatal complications associated with extradural block 0.42 per 10,000 maternities in the UK (95% CI 0.34 to 0.51) [30]. Failed tracheal intubation 0.38 per 10,000 maternities in the UK (95% CI 0.29 to 0.49) [31]. Deaths due to anaesthetic complications in the UK in 2010–12 0.02 per 10,000 maternities in the UK (95% CI 0.01 to 0.04) [23]	1.90	Comparable with the UK
Obstetric embolism (including Amniotic Fluid Embolism)	O88 (O88.0, O88.1, O88.2, O88.3, O88.8)	1742	2.73 (2.60 to 2.86)	Amniotic Fluid Embolism 0.17 per 10,000 maternities in the UK (95% CI 0.14 to 0.21) [32]. Antenatal pulmonary embolism 1.3 per 10,000 maternities in the UK (95% CI 1.1 to 1.5) [33]. Venous thromboembolism 8.5 per 10,000 maternities in a London hospital (95% CI 7.6 to 9.4) [34]	3.88	Not comparable
Shock	R57.0, R57.1, R57.2, R57.8, R57.9, O75.1, T80.5, T88.6	1252	1.96 (1.85 to 2.07)	Septic shock 0.91 per 10,000 maternities in the UK (95% CI 0.71–1.15). Incidence rates for septic shock in the UK were available from a UKOSS study that collected data from June 2011 to May 2012 [21]. Specific incidence of shock due to other complications during pregnancy and childbirth including those related to thromboembolism, AFE, etc could not be found.	6.41	Not comparable
Sickle cell anaemia with crisis	D57.0	291	0.46 (0.41 to 0.51)	0.68 per 10,000 maternities in the UK (95% CI 0.51 to 0.88)—calculated from a UK wide epidemiological study by Oteng-Ntim et al., 2015 [35]	Not reported	Comparable with the UK
Status asthmaticus	J46	142	0.22 (0.19 to 0.26)	Not available	0.42	Comparable with the Australian indicator
Status epilepticus	G41, G41.0, G41.1, G41.2, G41.8, G41.9	173	0.27 (0.23 to 0.31)	Not available	0.38	Comparable with the Australian indicator
Uterine rupture	O71.0, O71.1	3077	4.82 (4.65 to 4.99)	1.9 per 10,000 maternities in the UK (95% CI 1.6 to 2.2) [25]	5.29	Comparable with the Australian indicator
Eclampsia	O15.0, O15.1, O15.2, O15.9	4568	7.15 (6.94 to 7.36)	2.7 per 10,000 births in the UK (95% CI 2.4 to 3.1) [22]	Not included in the Australian indicator	Not comparable
Sepsis	O85	2803	4.39 (4.23 to 4.55)	4.7 per 10,000 maternities in the UK (95% CI 4.2–5.2) [21]	Not included in the Australian indicator	Comparable with the UK
Cerebral venous thrombosis	O87.3	16	0.03 (0.01 to 0.04)	Cerebral venous thrombosis 0.02 per 10,000 maternities in the UK (95% CI 0.003 to 0.04) [29]	Not included in the Australian indicator	Comparable with the UK
**Procedures indicating morbidity (OPCS 4.7 codes)**						
Assisted ventilation including tracheostomy	E85.1, E85.2, E42.1, E42.2, E42.3, E42.8, E42.9	978	1.53 (1.44 to 1.63)	Not available	2.64	Not comparable
Curettage in combination with a general anaesthetic	R28.1 + Y80	78	0.12 (0.01 to 0.15)	Not available	3.10	Not comparable
Dialysis	X40, X41.1, X42.1	92	0.14 (0.11 to 0.18)	Not available	0.34	Comparable with the Australian indicator
Evacuation of haematoma	P09.3, P27.1, T34.1, T34.2, T34.3, T45.1, T45.2, T45.3, T45.4, Y22.1	3,186	4.99 (4.82 to 5.16)	Incidence rate not available, but 3 cases of haematoma/ abscess were noted in the study of non-fatal anaesthetic complications in the UK by Scott and Hibbard, 1990[30]	6.11	Not comparable
Hysterectomy	Q07.1, Q07.2, Q07.3, Q07.4, Q07.5, Q08	1,507	2.36 (2.24 to 2.48)	Emergency peripartum hysterectomy due to any cause 4.8 per 10,000 maternities in a London hospital (95% CI 2.7 to 8.0) [36]Peripartum hysterectomy for management of severe obstetric haemorrhage 3.6 per 10,000 maternities in a Nottingham hospital (95% CI 2.1 to 5.7) [37]. Peripartum hysterectomy for management of severe obstetric haemorrhage 3.8 per 10,000 maternities in the UK (95% CI 3.3 to 4.2) [38]	3.12	Comparable with the UK and Australian indicator
Procedures to reduce blood flow to uterus	L69.3, L69.4, L70.2, L70.3, L71.3, L93.3, L94.1, L94.6, L94.7, L99.1, L99.5, L99.6	390	0.61 (0.55 to 0.67)	2nd line treatment for control of PPH (uterine compression sutures/ pelvic vessel ligation/ interventional radiology/ received rFVIIa). 2.2 per 10,000 maternities in the UK (95% CI 1.9 to 2.5) [39]. Rates recalculated after removing the 31 cases that received rFVIIa = 2.0 (95% CI 1.8 to 2.3) [39]	Embolisation or ligation of blood vessels = 0.80, Other interventions to control post-operative bleeding = 0.96	Comparable with the Australian indicator
Re-closure of disrupted caesarean section wound	T28.3, T30.1, T30.2, T30.3, T30.4, S42.3, S42.4, S60.4	1989	3.11 (2.98 to 3.25)	Not available	0.48	Not comparable
Repair of bladder or cystostomy	M37.2, M37.3, M37.5, M37.8, M37.9, M38.2, M38.3, M73.6, M73.7	1,991	3.12 (2.98 to 3.26)	Accidental cystostomy rate in Aberdeen Maternity hospital 1.4 per 10,000 maternities (95% CI 0.8 to 2.2) [40]	Repair of bladder = 2.26, Cystostomy = 1.28	Comparable with the Australian indicator
Repair of intestine	G58, G69, G70, G78, H06, H07, H08, H09, H10, H11, H23, H26, H29, H33, T37.4, T38.4, T42.1	48	0.008 (0.006 to 0.01)	Not available	0.36	Not comparable

N = 6,389,066; *95% Confidence intervals are included where available. ICD-10: International Statistical Classification of Diseases and Related Health Problems—10^th^ revision; OPCS: Classification of Interventions and Procedures, version 4.7 (April 2014)

## Discussion

Using routine hospital data on episodes of childbirth in England from April 2003 to March 2013 we were able to generate a composite indicator, EMMOI, to measure maternal morbidity outcomes during childbirth in England which included 26 morbid events (17 diagnoses and 9 procedures). Selection of individual morbid events was driven by the quality of the routine hospital data, which was variable and thus necessitated a number of adaptations. The composite indicator showed an increase in maternal morbidity outcomes during childbirth in England across the 10 years examined, with a 29% overall absolute increase in incidence. However, questions on reliability of the indicator remain due to mismatches between the rates of individual morbid events and the incidence of these conditions in the UK reported by population-based studies.

Comparing the overall rate of the individual component diagnoses and procedures included in the EMMOI with incidence of these morbid events in the UK reported by population-based studies and with the rates of the individual components of the Australian indicator estimated in the Australian population health datasets [41], showed considerable inconsistency (Table 3). Rates of six out of the 17 component diagnoses, cardiac arrest/ failure or infarction, cerebrovascular accident, major complications of anaesthesia, sickle cell anaemia with crisis, sepsis, and cerebral venous thrombosis, were comparable with the incidence of these conditions in the UK reported in the literature. Of the nine procedures included in the EMMOI only the rate of hysterectomy was comparable with the reported incidence rates for the UK. Rates of 10 morbid events were comparable with the Australian indicator (Table 3). Estimated incidence rates for 12 morbid events during childbirth in the UK were not available from existing studies: acute abdomen, acute renal failure, cerebral oedema or coma, disseminated intravascular coagulopathy, status asthmaticus, status epilepticus, assisted ventilation including tracheostomy, curettage in combination with general anaesthesia, dialysis, evacuation of haematoma, re-closure of disrupted caesarean section wound, and repair of intestine. We thus cannot rule out the possibility of both false positive and false negative cases which reduces the validity and reliability of the EMMOI.

Irrespective of the variations in definitions and indicators used to measure maternal morbidity, several studies from across the world show an increase in the incidence of maternal morbidity outcomes [41–43]. The observed increase in maternal morbidity outcomes during childbirth in England from 2003 to 2013 was mainly driven by an increase in sepsis, uterine rupture, assisted ventilation including tracheostomy and repair of bladder or cystostomy, although all morbid events showed a static or increasing trend except eclampsia.

The incidence of sepsis in the UK between 1 June 2011 and 31 May 2012 was estimated as 4.7 per 10,000 maternities [21] and confidential enquiries into maternal deaths in the UK showed an overall decrease in the rates of genital tract sepsis, but with considerable fluctuations across the years from 1985–87 to 2009–11 [23]. Although a decreasing trend in eclampsia in this study conforms to the findings of the Confidential Enquiry into Maternal Deaths which suggest that deaths due to hypertensive disorders during pregnancy (pre-eclampsia and eclampsia) are at the lowest ever recorded [23], the routine hospital data showed higher rates of eclampsia compared to that estimated by a previous UK wide epidemiological study (2.7 cases per 10,000 births) [22]. This could be due to coding errors in the HES data as discussed below.

The HES data includes diagnosis of acute psychosis among women during the childbirth episode (0.53; 95% CI 0.48 to 0.59), which would be expected to be lower than the overall incidence of acute psychosis in the UK (10 per 10,000 women) [26] as this condition generally occurs during the postnatal period after discharge. We were not able to find any other comparable data. Compared to the incidence of uterine rupture calculated from the HES data, the low uterine rupture rates measured by the UK Obstetric Surveillance System (UKOSS) could be due to the exclusion of women in whom an incidental asymptomatic uterine dehiscence was noted at caesarean section [25]. These cases are likely to have been classified as uterine rupture in the HES data.

Incidence rates of 10 out of 26 morbid events constituting the EMMOI were in agreement with the rates of the individual morbid events included in the Australian indicator estimated in the Australian population health datasets [41]. Incidence rates for three conditions, disseminated intravascular coagulopathy, cerebral oedema or coma, and sickle cell anaemia with crisis were not reported in the study by Roberts et al [41] that estimated the trends in maternal morbidity using the Australian indicator despite these being valid components of the Australian indicator [19]. It is not known whether this was due to lack of cases in the data or unreliability of the routine data used in the study. Retaining only the components that agree with the incidence figures in the UK or with the rates of the individual components of the Australian indicator would exclude 11 conditions from the English indicator, including eclampsia, obstetric embolism and cardiac arrest/ failure, which are associated with increased risk of maternal mortality in the UK [44]. The resultant composite indicator (EMMOI) would therefore be questionable in its ability to estimate accurately and reliably the incidence of maternal morbidity during childbirth in England.

### Strengths and limitations of this study

This study was an attempt to create a composite indicator to measure maternal morbidity outcomes during childbirth using routine hospital data from 6,389,066 women giving birth in England from 2003 to 2013. Although we were able to demonstrate that a composite indicator to measure maternal morbidity outcomes during childbirth can be constructed using routine hospital data, we were not able to validate the accuracy of the composite indicator in classifying women as having suffered a morbidity event, since data protection and privacy laws allowed only for access to anonymised data. The use of a composite measure helps to avoid or reduce concerns about false negatives, but we cannot exclude the possibility of false positives.

It is important to acknowledge the limitation of not being able to use the data on blood transfusion, PPH and ‘repair of ruptured or inverted uterus’ which we did not find to be reliable. While the information collated from the NHS hospitals goes through a process of cleaning and quality checks, the quality of routine data collected by the HSCIC including HES has been questioned by a number of audits [45–47]. It has been noted that in most hospitals the diagnoses and procedures are coded by trained clinical coders from the hospital notes which are not structured or standardised, as a result errors and omissions occur [47]. In addition to errors in the data, we cannot rule out the play of chance, bias and confounding due to variations in clinical practice and the definitions of the morbid conditions used across the NHS hospitals. This substantially compromises the validity and reliability of the incidence estimates of maternal morbidity during childbirth in England measured using the EMMOI.

Beyond the coding limitations we describe, it is difficult to determine the reasons for the observed differences in codes and procedures relating to uterine rupture and postpartum haemorrhage. We could speculate that procedures, particularly where these require an operation under anaesthesia, such as repair of ruptured uterus, are more reliably coded than diseases/disorders when codes could be included in error simply from a list of differential diagnoses. Similarly, since blood transfusion is a more clearly defined procedure, requiring detailed checking, it may be more accurately recorded than postpartum haemorrhage; estimation of blood loss post-delivery is known to be inaccurate [48]. Conversely, blood transfusion is recorded in other databases, and it is possible that when the record of the transfusion is included in the National Health Service (NHS) Blood and Transplant database, it is less likely to be recorded in Hospital Episode Statistics. These databases are not linked, and therefore we are unable to assess whether this is the case.

## Conclusion

A single measure of maternal morbidity could be a reliable indicator of quality of pregnancy care in some settings, and an indicator which uses routine data could be a cost-effective tool to monitor the quality of pregnancy care in England. However, while our study showed that routine English hospital data can be used to generate a composite indicator to monitor trends in maternal morbidity outcomes during childbirth, the quality and reliability of this monitoring indicator is dependent on the quality of the hospital data. Using the available data, we found that some codes were unusable due to major concerns about their validity. We could not confirm the reliability of the indicator due to mismatches between the rates of the individual components of the EMMOI calculated using the HES data and the rates reported by population-based epidemiological studies in the UK. The ongoing efforts to improve the reporting and quality of NHS hospital data [49], could make this a valuable resource to measure incidence and trends of maternal morbidity outcomes in England, but currently the data are not sufficiently robust to do so.
